# Enhanced Indoor Mobile Robot Localization via Lie-Group IMU–UWB Fusion and Dual-Stage Kalman Filtering

**DOI:** 10.3390/s26092686

**Published:** 2026-04-26

**Authors:** Zhengyang He, Xiaojie Tang, Muzi Li, Fengyun Zhang

**Affiliations:** 1 School of Intelligent Manufacturing, Sichuan University Jinjiang College, Meishan 620860, China; hezhengyang@scujj.edu.cn (Z.H.); tangxiaojie@scujj.edu.cn (X.T.); 2 Google Information Technology (USA) Co., Ltd., Seattle, WA 98103, USA; muzil@google.com; 3 College of Artificial Intelligence, Southwest University, Chongqing 400715, China

**Keywords:** robot localization, Lie group, Lie algebra, IMU/UWB, Kalman filter

## Abstract

Indoor mobile robots often experience degraded localization accuracy and robustness when relying on a single positioning modality. In addition, conventional pose computation based on Euler-parameterized transformations can be computationally involved and susceptible to singularities, while practical fusion schemes may not adequately suppress measurement errors. This paper proposes an indoor robot localization method, termed IMU_UWB_ESKF, which tightly fuses inertial and UWB measurements using a Lie-group state representation. IMU- and UWB-derived quantities are formulated on the associated Lie algebra, enabling numerically stable pose propagation and measurement updates. To mitigate sensor noise and reduce drift, a dual-stage Kalman filtering strategy is adopted: an EKF-based measurement correction is first performed, followed by a multi-dimensional error-state Kalman filter for refined fusion. The proposed pipeline is implemented on a wheeled-robot platform under ROS, integrating real-time IMU/UWB parameter extraction, pose transformation, and online state estimation. Experimental results demonstrate stable real-time localization with improved robustness and accuracy under dynamic motion, indicating the method’s applicability to indoor navigation tasks.

## 1. Introduction

Indoor localization is a fundamental capability for mobile robots and location-based services (LBS), underpinning navigation, tracking, and context-aware applications in smart buildings and the Internet of Things. The demand for reliable LBS continues to grow across a wide range of scenarios, including operation in hazardous environments with unknown maps, robot localization in highly structured warehousing systems, airport ground services for automated luggage handling, and monitoring robots deployed in non-geometric, dynamically changing agricultural settings such as commercial poultry farms [1,2,3,4]. Compared with outdoor environments, indoor spaces are characterized by severe multipath, frequent non-line-of-sight (NLOS) propagation, and dynamic occlusions from walls, furniture, and pedestrians. These factors impose stringent requirements on sensor reliability, estimator stability, and real-time performance.

A variety of indoor localization technologies have been investigated, including LiDAR [5], ultra-wideband (UWB) [6], and WiFi [7]. Each modality entails distinct trade-offs in achievable accuracy, deployment cost, and sensitivity to environmental conditions. Among them, UWB has attracted substantial attention due to its fine time resolution and practical potential for decimeter- to centimeter-level positioning under favorable conditions. However, UWB measurements are often biased or intermittently unreliable under NLOS and strong multipath, which can substantially degrade accuracy and robustness.

To enhance UWB-based localization, prior work has explored both measurement-level and system-level advances, including two-way ranging (TWR) and double-sided TWR (DS-TWR) variants [8], time of arrival (ToA) and time difference of arrival (TDoA) [9,10], angle of arrival (AoA), and received signal strength (RSS)-based methods [11]. In addition to ToA-based ranging, TDoA has also been widely studied in indoor UWB localization. Its practical advantages in anchor-based deployments have motivated substantial research on TDoA-based indoor positioning, including estimator design, robustness enhancement, and public benchmark datasets. For this reason, the TDoA-based UWB positioning module adopted in this work is consistent with existing indoor localization literature. In parallel, multi-sensor fusion has become a principal route to improving robustness: an inertial measurement unit (IMU) provides high-rate motion propagation, while UWB offers absolute geometric constraints to bound drift [12,13,14]. Extended Kalman filter (EKF)-based IMU–UWB fusion remains widely adopted due to its computational efficiency and relatively low deployment overhead [15,16]. Nevertheless, practical deployments commonly encounter three persistent challenges. First, UWB outliers and biased measurements under NLOS can induce filter inconsistency and accumulated drift. Second, pose representation and update using conventional parameterizations may lead to numerical fragility (e.g., singularities) during aggressive or prolonged motion. Third, achieving reliable end-to-end real-time performance requires stable preprocessing, coordinate transformations, and fusion logic; otherwise, error accumulation and unstable state updates may occur. Recently tightly-coupled fusion methods and learning-based localization frameworks have attracted more and more attention from researchers. Huang, Zexia proposed a novel hybrid fusion framework that combines the Extended Kalman Filter (EKF) and Recurrent Neural Network (RNN) to address challenges such as sensor frequency asynchrony, drift accumulation, and measurement noise [17]. The EKF provided real-time statistical estimation for initial data fusion, while the RNN effectively models temporal dependencies, further reducing errors and enhancing data accuracy. A deep learning (DL) based approach for near-field (NF) source localization is introduced to enhance the accuracy and robustness in both direction of arrival (DOA) and range estimation [18].

From the perspective of state estimation, conventional IMU–UWB fusion based on EKF-type frameworks is already well established. Therefore, the contribution of this work is not the general concept of IMU–UWB fusion itself, but the specific integration of three components into a practical indoor localization pipeline: (i) TDoA-based UWB positioning, (ii) EKF-based stabilization of UWB measurements, and (iii) Lie-group error-state fusion with IMU using an ESKF. The relevance of Lie-group/Lie-algebra tools lies in the fact that rigid-body pose naturally evolves on a nonlinear manifold, whereas uncertainty can be more consistently represented as a small perturbation in the tangent space. This formulation improves the numerical stability of state propagation and correction, avoids singularities associated with non-minimal attitude parameterizations, and is particularly suitable for real-time motion estimation with inertial sensing.

This paper proposes an indoor localization pipeline termed IMU_UWB_ESKF to address these issues through a staged estimation design. First, a TDoA-based UWB positioning module produces raw position measurements, which are subsequently smoothed using an EKF to yield a more stable UWB_EKF output. Second, IMU-based state propagation and UWB_EKF observations are fused using an error-state Kalman filter (ESKF) formulated with Lie-group/Lie-algebra tools. Specifically, the nominal state evolves on the rigid-body motion manifold, while uncertainty is modeled and estimated in the tangent space, enabling stable propagation and correction. This design implements the dual-stage Kalman filtering strategy adopted in this work: an EKF is applied to UWB measurements, followed by Lie-group ESKF fusion with IMU to produce the final IMU–UWB–ESKF trajectory.

The main contributions of this paper are summarized as follows:

- We propose a robust IMU–UWB fusion scheme that integrates TDoA-based UWB positioning with a dual-stage Kalman filtering framework (UWB_EKF and Lie-group IMU_UWB_ESKF), improving localization reliability in obstructed indoor environments.
- We introduce a Lie-group error-state formulation for stable pose estimation. By exploiting the perturbation structure of the ESKF and performing updates in the tangent space, the proposed method avoids singularities in rotation representation and improves numerical robustness.
- We implement the proposed algorithm on a ROS-enabled wheeled robot and conduct experiments in both open and obstacle-rich environments. In this experiment, the laser measurement is taken as the true value, and the measurement accuracy of the three methods is counted and the kinematic parameters are compared.

The rest of the paper is organized as follows. Section 2 reviews related work on indoor localization modalities, UWB positioning and NLOS handling, IMU–UWB fusion, and Lie-group filtering. Section 3 presents the system model and problem formulation. Section 4 details the UWB_EKF module, the Lie-group ESKF fusion method and an accurate reference system with laser range. Section 5 describes the experimental implementation. Section 6 reports experimental results and analysis. Section 7 concludes the paper.

## 2. Related Work

Indoor localization spans a spectrum from infrastructure-based to infrastructure-light solutions, including UWB, WiFi fingerprinting, and Bluetooth, where practical selection is typically driven by accuracy, deployment cost, and robustness to environmental variability. UWB positioning most commonly relies on time- and/or angle-based measurements—such as time of arrival (ToA), time difference of arrival (TDoA) [19], and angle of arrival (AoA)—but its performance can be severely impaired by non-line-of-sight (NLOS) propagation, which introduces systematic bias and increases measurement uncertainty [20,21]. Accordingly, substantial effort has been devoted to detecting, identifying, and mitigating NLOS effects in indoor UWB systems. or TDoA localization, algorithmic refinements often focus on improving estimator robustness under measurement noise and unfavorable anchor geometry. Weighted optimization strategies have been proposed to enhance TDoA accuracy by accounting for both stochastic noise characteristics and geometric dilution effects [22]. Iterative multi-stage adaptive estimation (MAE) methods further provide robust, closed-form or near closed-form solutions while balancing computational efficiency against communication overhead in networked settings [23]. Beyond time-based ranging, RSS-based localization and tracking has also been studied; for example, successive weighted RSS projection schemes aim to increase stability and accuracy when RSS measurements are noisy or temporally fluctuating [24].

NLOS mitigation has been approached from both model-based and data-driven perspectives. A representative classical strategy is to fuse inertial navigation with UWB and employ hypothesis testing (e.g., using yaw-related cues) to reject or down-weight NLOS-contaminated observations [25]. More recently, deep learning has been leveraged to correct fusion residuals and to learn robust representations under multipath and occlusion. Transformer-based architectures have been explored for compensating UWB/IMU fusion errors [26], and graph neural networks have been used to capture spatiotemporal dependencies for robust localization under complex indoor dynamics [27]. These advances are supported by survey studies that synthesize key challenges and research directions [28], as well as public TDoA datasets that facilitate training, evaluation, and benchmarking [29]. In addition, deep models have been applied directly to UWB signal-level inference, including LOS/NLOS classification via transfer learning to improve generalization across environments [30]. Complementary sensing can further provide auxiliary constraints in NLOS conditions; for instance, WiFi can be integrated with UWB to increase observability and reduce failure modes when UWB coverage deteriorates [31].

Related developments in indoor positioning and state estimation extend beyond UWB-centric pipelines. Examples include wireless body area network platforms for indoor positioning and motion tracking (e.g., “Zyggie”) [32], enhancements to EKF-based monocular SLAM that improve observability and reduce drift by incorporating attitude, altitude, and range-to-base information [33], hybrid systems combining visible-light proximity detection with Bluetooth RSS trilateration [34], and online multi-feature adaptive optimization for LiDAR–GPS/INS extrinsic calibration with validation on KITTI and real-world datasets [35]. Collectively, these studies underscore the diversity of sensing modalities and estimator designs, as well as the persistent tension between deployment complexity and robustness in realistic environments. To improve robustness while maintaining low deployment overhead, IMU–UWB fusion has been widely adopted, leveraging the IMU for high-rate motion propagation and UWB for absolute geometric constraints. Particle-filter formulations, such as dynamic feasible region-based particle filters (DFRPF), have been proposed to improve particle convergence and enhance robustness under NLOS [36]. Tightly coupled IMU/UWB navigation methods that incorporate carrier motion characteristics have also been reported to improve consistency during dynamic maneuvers [37]. Beyond UWB, IMU fusion with Bluetooth has been investigated to achieve sub-meter indoor positioning accuracy in cost-sensitive configurations [38]. Robust Bayesian filtering variants have been introduced as well, including Monte Carlo-based robust unscented Kalman filters (MC-RUKF) with adaptive kernel bandwidth to address limitations of conventional Monte Carlo filtering in low-cost UWB/IMU setups [39]. For UWB NLOS handling, learning-based classification and recognition algorithms—such as SOM-based batch processing and CNN-based recognition—have been developed to identify NLOS conditions and reduce their impact on estimation [40]. While EKF-based fusion remains a common baseline, more advanced formulations—including the error-state Kalman filter (ESKF) and Lie-group-based filters (e.g., IEKF) on SO(3)/SE(3)—often provide improved numerical stability by estimating perturbations in the tangent space and evolving nominal states on the manifold [41].

More advanced formulations have therefore been developed to improve numerical consistency and robustness, including error-state Kalman filtering and Lie-group-based filtering on SO(3)/SE(3). In these methods, the nominal state evolves directly on the motion manifold, while the estimation error is modeled in the associated tangent space. This separation is advantageous for inertial navigation and pose estimation because it preserves the geometric structure of rigid-body motion and enables locally minimal error representations. In this manuscript, we adopt this line of methodology not as a completely new filtering theory, but as a practically motivated formulation for stable IMU–UWB fusion in indoor robot localization. Motivated by these observations, this manuscript adopts a cascaded filtering design: UWB measurements are first stabilized via an EKF, and a Lie-group ESKF then performs tightly coupled IMU–UWB fusion to achieve accurate and stable real-time localization under NLOS and dynamic conditions.

Positioning of this work: In contrast to approaches that rely on a single modality or introduce additional sensors that increase deployment and calibration complexity, this study targets a low-cost IMU–UWB configuration and integrates (i) TDoA-based UWB positioning, (ii) EKF-based UWB stabilization, and (iii) Lie-group error-state fusion for real-time robot localization. The method is validated in both open-area and occlusion-rich scenario. Finally, the error statistical analysis of the three measurement methods is completed.

## 3. System Design

**Figure 1:**
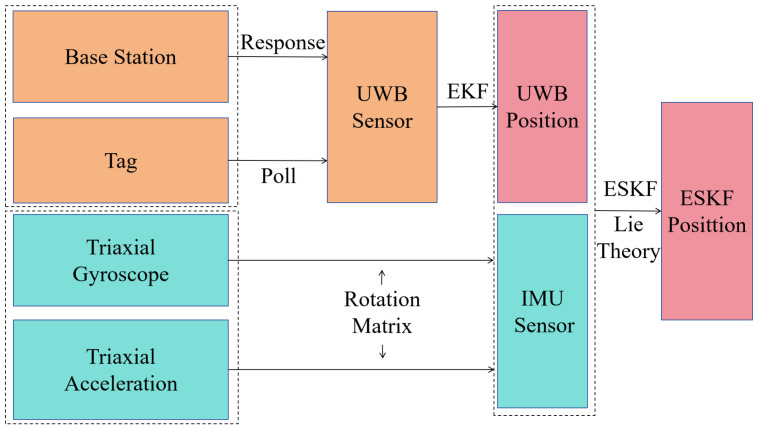
System framework of the scheme. Different colors indicate different functional modules, and the dotted frame denotes the IMU–UWB fusion and state-estimation module.

The overall framework of the proposed mobile localization algorithm, termed IMU_UWB_ESKF, is illustrated in Figure 1. IMU and UWB positioning are integrated by combining Lie-group/Lie-algebra modeling with Kalman filtering applied in two stages. First, an EKF is used to process UWB measurements and generate UWB_EKF. Then, an ESKF fuses IMU propagation with UWB_EKF to obtain the final IMU_UWB_ESKF result. Lie-group theory is adopted because it provides a robust pose representation and avoids singularities compared with Euler angles, quaternions, or direct rotation-matrix parameterization, by evolving the nominal state on the manifold and estimating errors in the tangent space. The dual-stage Kalman design is motivated by IMU drift from time-accumulated errors and the susceptibility of UWB to NLOS-induced outliers and bias; therefore, a UWB-based EKF and a Lie-group-based ESKF are jointly employed in the proposed framework.

The core process of this system is as follows: The system adopts a loosely coupled UWB and IMU fusion architecture to realize high-precision positioning: firstly, a base station and a tag in a UWB subsystem complete ranging through a response-polling mechanism, and raw data is filtered by an EKF to obtain preliminary UWB position estimation; meanwhile, a three-axis gyroscope and a three-axis accelerometer in the IMU subsystem respectively perform coordinate conversion and data fusion through a rotation matrix to generate IMU sensor information; finally, the UWB position and IMU data are input into the ESKF filter based on Lie group theory, and the optimal fusion of the two sensors is realized by using Lie algebra to deal with the nonlinear characteristics of rotation, and the accurate and reliable ESKF final position estimation is output.

## 4. UWB_EKF and Lie-Group ESKF Fusion Method

### 4.1. The Basis of IMU Mobile Positioning

The mobile robot is equipped with an IMU that outputs tri-axial acceleration and angular velocity measurements. Given initial conditions, the robot state can be propagated in real time by integrating the gyroscope readings to update attitude and integrating the (gravity-compensated) specific force to update velocity and position. For example, from time *i* to time *j*, the state can be propagated by accumulating only the intermediate IMU measurements as follows.

(1) $$\begin{array}{c} \left\{\begin{array}{c} R_{j}\;=\;R_{i}\prod_{k=i}^{j-1}\mathrm{Exp}\;\left(({\tilde{\omega }}_{k}\;-\;b_{g,k})\;\Delta t\right),\\ p_{j}\;=\;p_{i}\;+\;\sum_{k=i}^{j-1}\left[v_{k}\;\Delta t\;+\;\frac{1}{2}g\;\Delta t^{2}\right]\;+\;\frac{1}{2}\sum_{k=i}^{j-1}R_{k}\left({\tilde{a}}_{k}\;-\;b_{a,k}\right)\Delta t^{2},\\ v_{j}\;=\;v_{i}\;+\;\sum_{k=i}^{j-1}\left[R_{k}\left({\tilde{a}}_{k}\;-\;b_{a,k}\right)\Delta t\;+\;g\;\Delta t\right]. \end{array}\right. \end{array}$$

where *R* represents the rotation part of the robot during the movement, which is a rotation matrix; $\tilde{\omega }$ represents the instantaneous angular velocity of the robot at a certain moment; $b_{g}$ and $b_{a}$ represent the zero bias of the gyroscope and the accelerometer respectively; and g represents the acceleration of gravity. $\tilde{a}$ represents the acceleration of the robot in the vehicle coordinate system, and *p* and *v* represent the position and velocity of the robot in the world coordinate system respectively. Subscripts *i*, *j* and *k* represent time instants, and *i* represents the previous time instant. Δt represents sampling time.

### 4.2. The Principle of UWB Positioning

**Figure 2:**
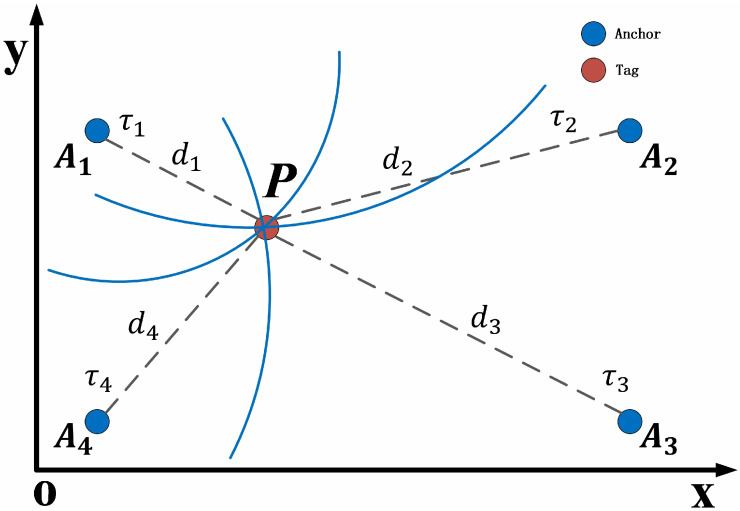
TDoA model of the project.

The UWB localization subsystem used in this work is built on the Qorvo DW1000 evaluation board (Qorvo, Inc., Greensboro, NC, USA) The DW1000 is a SoC system solution with ranging accuracy within 30 cm. It has the advantages of small size, low cost, and anti-multipath interference. The TDoA ranging model is used in the project, and the measurement schematic diagram of the model is illustrated in Figure 2.

In the project, the positioning system deploys four anchor nodes in the environment, namely $A_{1}$, $A_{2}$, $A_{3}$, and $A_{4}$. The coordinate values of all anchor nodes are measured in a reference coordinate system, and a coordinate value of the Anchor node *i* is recorded as $\left(x_{i},\;y_{i},\;z_{i}\right)$. The coordinate of the Tag node *P* is unknown, marked as (x, y, z). The distance measurement value between the Tag node and the anchor node *i* is marked as $d_{i}$. The position coordinates of the target point can be obtained by using the minimum mean square error estimation as follows.

(2) $$\begin{array}{c} \left[\begin{array}{c} x\\ y\\ z \end{array}\right]\;=\;-\frac{1}{2}\;{\left(A^{T}A\right)}^{-1}A^{T}\left(\left[\begin{array}{c} d_{1}^{2}\;-\;d_{n}^{2}\\ d_{2}^{2}\;-\;d_{1}^{2}\\ \vdots \\ d_{n-1}^{2}\;-\;d_{n}^{2} \end{array}\right]-c\right) \end{array}$$

where *A* is the difference between the coordinates of each anchor point and *c* is a constant associated with each anchor coordinate.

**Figure 3:**
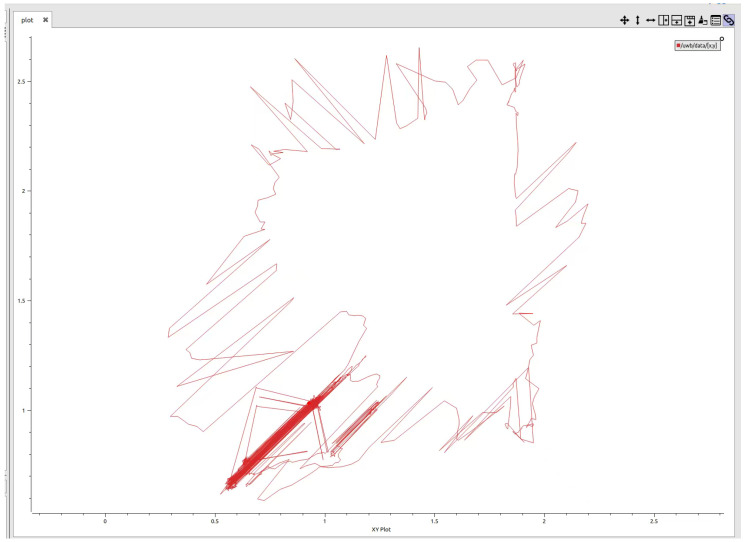
UWB anchor layout and planned route for mobile robot.

Positioning noise of UWB at rest can be seen in Figure 3. The drift of positioning error of pure UWB is up to 0.5 m at rest, and it is obviously reduced to about 0.2 m after ESKF filtering in the subsequent fusion results. Therefore, the IMU–UWB–ESKF framework proposed in this paper is generally sensitive to the measurement, and mainly integrates the motion process variables and the basic UWB positioning through the idea of Lie group.

### 4.3. UWB Location Model Based on Extended Kalman Filter (EKF)

The UWB positioning calculation is a nonlinear equation, so the EKF algorithm of the nonlinear system is used to deal with the UWB positioning parameters as follows.

(3) $$\begin{array}{c} \left\{\begin{array}{cc} K_{k} & \;=\;P_{k,\mathrm{pred}}\;H_{k}^{T}{\left(H_{k}\;P_{k,\mathrm{pred}}\;H_{k}^{T}\;+\;Q_{k}\right)}^{-1},\\ x_{k} & \;=\;x_{k,\mathrm{pred}}\;+\;K_{k}\;\left(Z_{k}\;-\;h\;\left(x_{k,\mathrm{pred}}\right)\right),\\ P_{k} & \;=\;\left(I\;-\;K_{k}H_{k}\right)P_{k,\mathrm{pred}}. \end{array}\right. \end{array}$$

where $K_{k}$ is the Kalman gain; $H_{k}$ is observation matrix; $Q_{k}$ is the covariance matrix of the observation noise; $P_{k}$ is the covariance matrix of the posterior estimation error; $x_{k}$ is posteriori state estimation; $x_{k,pred}$ is last state estimation; $Z_{k}$ is actual observation vector; $h(x_{k,pred})$ is predicted observations; $P_{k,pred}$ is covariance matrix of prior estimation error; and *I* is the identity matrix.

### 4.4. UWB and IMU Fusion Localization Model Based on ESKF

In this project, UWB measurements are first filtered by an EKF, after which IMU–UWB fusion is performed using an ESKF. Compared with a conventional EKF, the ESKF offers several advantages. During rotation estimation, the ESKF represents attitude errors with a minimal three-parameter perturbation, i.e., a three-dimensional increment defined in the tangent space (a vector space). As the error state is maintained near the origin, singularities are avoided and numerical stability is improved. Moreover, because the error-state variables remain small, higher-order terms are typically negligible; consequently, the associated Jacobians become simpler and, in some cases, can be approximated by identity matrices.

The ESKF procedure used in this project is summarized as follows. When IMU measurements arrive, they are integrated to propagate the nominal state (i.e., the main state estimate). Since this propagation does not explicitly correct for noise and bias at each step, drift accumulates over time. Therefore, an error state is introduced to model the deviation between the nominal state and the true state, and its evolution is driven by approximately Gaussian process noise. The mean and covariance of this error state characterize the uncertainty growth during motion. To correct the drift, the ESKF update relies on external observations beyond the IMU; in this work, the stabilized UWB output (UWB_EKF) is used. During the update, the posterior mean and covariance of the error state are computed from the measurement residual. The estimated error is then injected into the nominal state, and the error state is reset to zero, completing one predict–update cycle. The error-state update can be computed as follows.

(4) $$\begin{array}{c} \left\{\begin{array}{c} K\;=\;P_{\mathrm{pred}}\;H^{T}{\left(H\;P_{\mathrm{pred}}\;H^{T}\;+\;V\right)}^{-1},\\ \delta x\;=\;K\left(z\;-\;h\;\left(x_{\mathrm{pred}}\right)\right),\\ x\;=\;x_{\mathrm{pred}}\;+\;\delta x,\\ P\;=\;\left(I\;-\;KH\right)\;P_{\mathrm{pred}}. \end{array}\right. \end{array}$$

where *K* is the Kalman gain; *H* is observation matrix; *V* is the covariance matrix of the observation noise; $P_{p}red$ is the covariance matrix of the prior estimation error; δx is the estimated optimal error state; $x_{p}red$ is last state estimation; $Z_{k}$ is actual observation vector; $h(x_{k,pred})$ is predicted observations; *P* is the covariance matrix of the posterior estimation error; and *I* is the identity matrix. The letter of the posterior estimate omits the following table *k*.

The parameters in the algorithmic formulation are determined according to the actual hardware configuration and field test conditions. Specifically, *F* denotes the state transition matrix of the Kalman filter, *P* denotes the covariance matrix, *H* denotes the Jacobian matrix, *Q* denotes the process noise matrix, and *R* denotes the measurement noise matrix. Their specific forms are given as follows:

(5) $$F\;=\;\left(\begin{array}{cccc} 1 & 0 & \frac{1}{30} & 0\\ 0 & 0 & \frac{1}{30} & 0\\ 0 & 0 & 1 & 0\\ 0 & 0 & 0 & 1 \end{array}\right)$$

(6) $$H\;=\;\left(\begin{array}{cccc} 1 & 0 & 0 & 0\\ 0 & 1 & 0 & 0 \end{array}\right)$$

(7) $$Q\;=\;\left(\begin{array}{cccc} 0.1 & 0.1 & 0.1 & 0.1 \end{array}\right)$$

(8) $$R\;=\;\left(\begin{array}{cc} 1 & 1 \end{array}\right)$$

The IMU noise parameters are further set as follows:

(9) $$\mathrm{imuAccNoise}\;=\;5.6163036720342360\;\times \;10^{-3}$$

(10) $$\mathrm{imuGyrNoise}\;=\;1.4930239157013774\;\times \;10^{-3}$$

(11) $$\mathrm{imuAccBiasN}\;=\;1\;\times \;10^{-4}$$

(12) $$\mathrm{imuGyrBiasN}\;=\;1\;\times \;10^{-6}$$

### 4.5. Core Composition of IMU_UWB_ESKF Positioning Algorithm

In order to completely and clearly describe the whole program flow of the mobile positioning scheme in the article, the core composition of the IMU_UWB_ESKF positioning algorithm is described in this subsection, which contains seven algorithmic steps.

### 4.6. An Accurate Reference System by Laser Range

**Figure 4:**
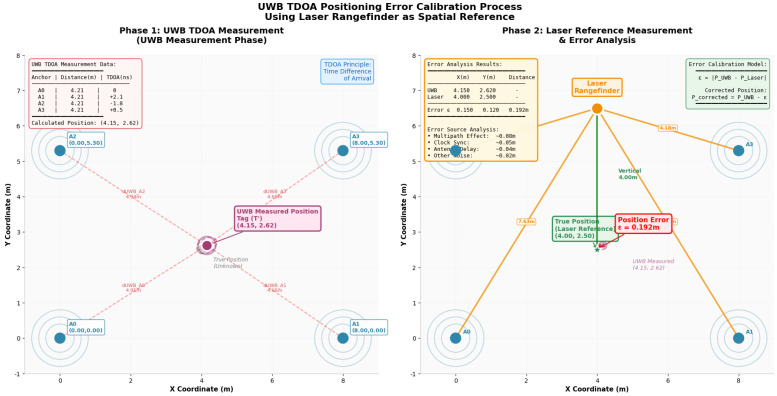
UWB positioning error calibration process by laser ranging.

In order to address the requirement for an accurate reference system, a laser range finder is utilized to establish the ground truth for spatial measurements. Figure 4 is the experimental diagram of the tag compared by UWB ranging and laser ranging. Through the experiment, it can be found that the error of UWB measurement data relative to the true value is 0.192 m, which is reasonable and in line with the ordinary measurement accuracy of UWB equipment. This lays the foundation for the reliability of multi-point measurement and calibration in the subsequent robot movement process.

## 5. The Experimental Implementation Process

### 5.1. Open Field Experiment

Verification experiments for the proposed algorithm are conducted in two indoor scenarios: an open area and an occlusion (sheltered) area. This subsection describes the open-area experiment.

The proposed localization framework is implemented on a ROS-enabled wheeled robot platform. The software pipeline consists of a UWB positioning node, an IMU data subscription node, a UWB-EKF node for measurement stabilization, and an ESKF-based fusion node for IMU–UWB integration. Experimental data are recorded through ROS (Open Source Robotics Foundation, Mountain View, CA, USA; https://www.ros.org/, accessed on 25 April 2026) and subsequently analyzed in MATLAB (MathWorks, Natick, MA, USA; https://www.mathworks.com/products/matlab.html, accessed on 25 April 2026), while the motion and estimated trajectories are visualized in RViz (Open Source Robotics Foundation, Mountain View, CA, USA; https://wiki.ros.org/rviz, accessed on 25 April 2026). This software structure allows real-time acquisition, filtering, fusion, and offline evaluation within a unified experimental workflow.

**Figure 5:**
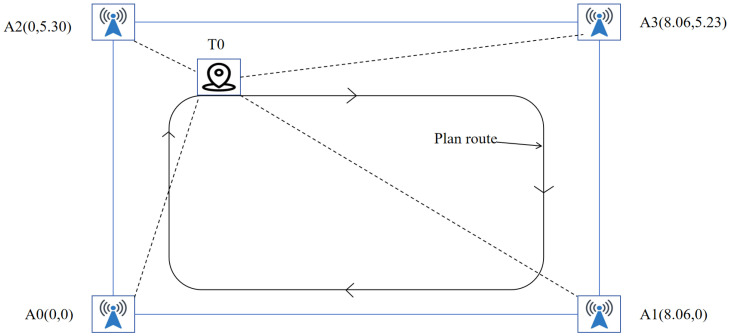
UWB anchor layout and planned route in the open-field experiment.

First, a TDoA-based UWB positioning program is implemented for the deployed UWB hardware, and an EKF module for UWB measurement smoothing is developed in Visual Studio Code (Microsoft Corporation, Redmond, WA, USA; https://code.visualstudio.com/, accessed on 25 April 2026). Subsequently, an IMU–UWB ESKF fusion module is implemented to generate localization coordinates for the mobile robot. Prior to the experiment, the coordinates of the four UWB anchors are measured using a tape measure and then entered into the UWB positioning program. The robot is equipped with an onboard IMU module that provides tri-axial acceleration and angular velocity measurements for inertial propagation. The IMU data stream is subscribed and processed in ROS at 50 Hz, while the UWB localization subsystem updates at 30 Hz. These two sensing streams are synchronized within the fusion framework to support real-time state estimation. In this project, the IMU output rate is set to 50 Hz, and the UWB system update rate is set to 30 Hz. The anchor coordinates are shown in Figure 5. The coordinate system is defined with A0 as the origin, and the planned trajectory of the mobile robot is specified accordingly. After initialization, the UWB tag is mounted above the robot platform, completing the experimental setup.

The experimental procedure is summarized as follows. First, the chassis drive node is enabled in the ROS system of the mobile robot. Data logging is then started, after which the UWB positioning node and the IMU parameter subscription node are launched. Next, the UWB-based EKF node is started. Finally, the fusion node based on UWB_EKF and IMU is launched. The robot is initially aligned with the positive X-axis and kept stationary for 30 s to allow the ROS system to complete IMU self-calibration. The robot is then commanded to follow the predefined trajectory. During the experiment, the mobile robot’s translational speed and steering angle are regulated to ensure repeatable motion.

**Figure 6:**
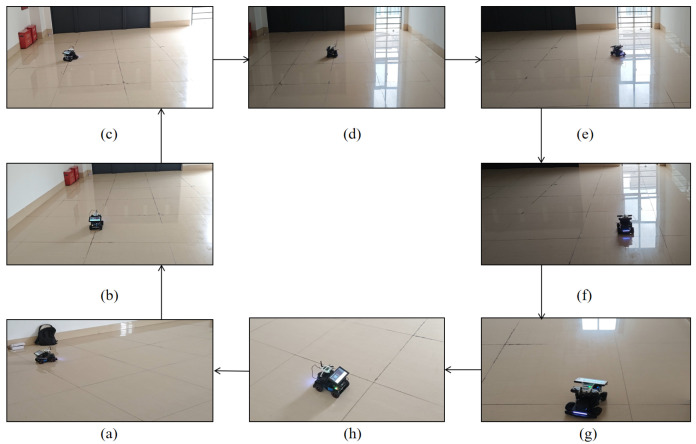
Robot navigation and positioning experiment without obstacles.(**a**) Starting point. (**b**) Midway point P3. (**c**) Midway point P4. (**d**) Midway point P5. (**e**) Midway point P6. (**f**) Midway point P8. (**g**) Turning point between P8 and P9. (**h**) Midway point P10.

The robot begins to move from the initial position of the planned route, as shown in Figure 6a After reaching a certain position, a right turn is initiated, followed by continued straight movement as presented in Figure 6c. Finally, a full loop around the field is completed, ending at the starting point. The data is then saved, and the program is closed. At this stage, the unobstructed mobile localization experiment of the mobile robot has been completed.

### 5.2. Experiment with Sheltered Field Inside

**Figure 7:**
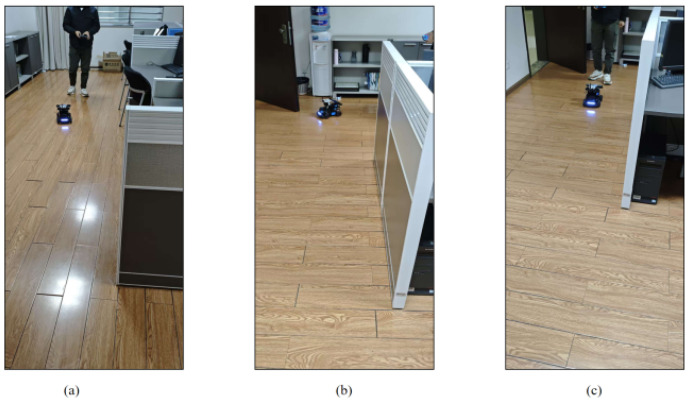
Localization experiment of robot with occlusion. (**a**) Midway point P1. (**b**) Midway point P4. (**c**) Midway point P6.

**Figure 8:**
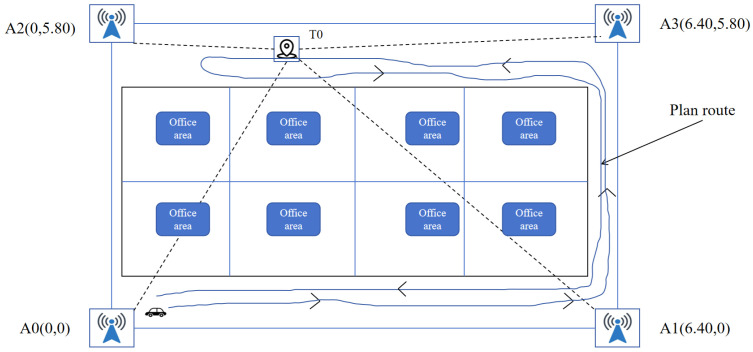
UWB anchor layout and robot planned route with obstacles.

After the open space experiment, in order to further analyze the performance of the algorithm, the indoor sheltered space experiment is carried out as shown in Figure 7. The test site is selected in the office, and the office area of the office is divided into a U-shaped long and narrow space. As in the previous preparations, after the system is ready, the next step is to drive the robot around the field according to the predetermined route as shown in Figure 8 and record the data.

## 6. Experiment Results and Analysis

### 6.1. Discussion of Positioning Experiment Without Obstacles

**Figure 9:**
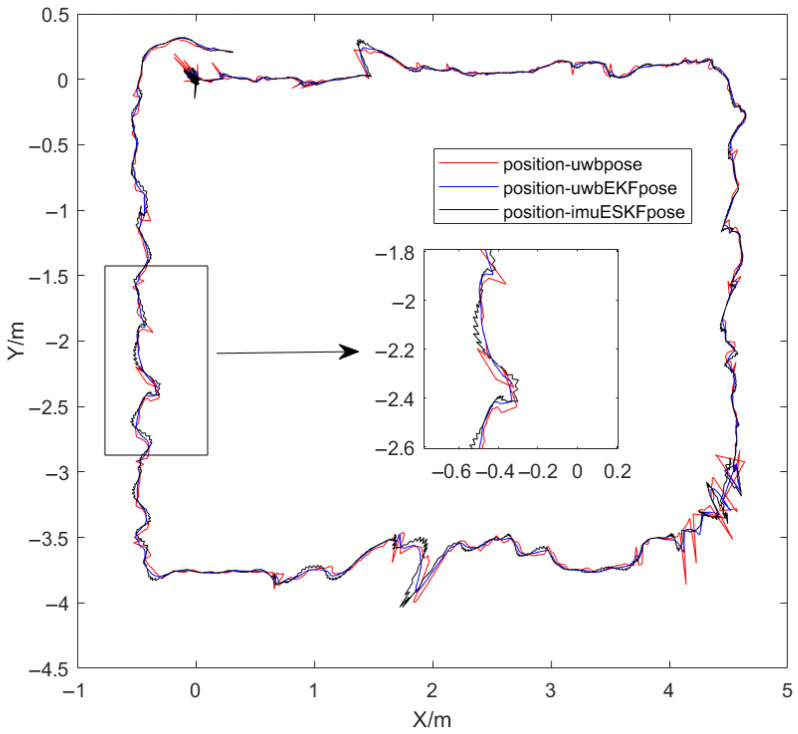
The visualization of the movement process.

Following the mobile robot’s positioning experiment, data were exported for analysis. With the UWB receiving frequencies set at 8 Hz, data were sequentially processed by the EKF and then the ESKF fusion algorithm. In MATLAB, the experiment is conducted within an approximately 5 m × 4 m rectangular area as shown in Figure 9. The red trajectory, from the UWB system alone, shows significant noise. The blue trajectory, representing EKF_UWB data, exhibits reduced sharp protrusions. A improvement clearly visible in the local inset confirmed effective filtering. The black trajectory, resulting from the ESKF_UWB_IMU, is notably smoother. However, it displays characteristic high-frequency, small-amplitude oscillations, result in incorporating the dynamic IMU measurements into the fusion process.

**Figure 10:**
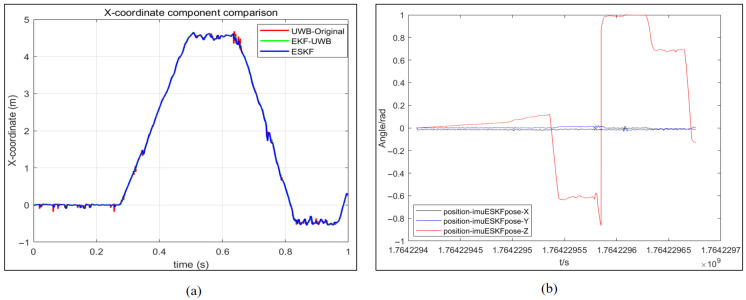
Analysis of robot motion parameter component. (**a**) X-coordinate comparison of mobile robots. (**b**) The change in the rotation angle of the XYZ-axis of the mobile robot under the ESKF positioning method.

A comparison of the X coordinates of the mobile robot under different positioning methods is presented in Figure 10a. From the figure, it can be observed that the curves obtained after the EKF and ESKF algorithms are largely coincident, while obvious bulges are present only in the X coordinate component of the original UWB positioning methods. The variation in the XYZ-axis rotation angles of the mobile robot under the ESKF positioning method is shown in Figure 10b. Since IMU parameters are only utilized in the sensor information fusion of the ESKF, the XYZ-axis rotation angles of the ‘position-imuESKpose-X’ only are displayed. It can be observed that the rotation angles around the X and Y axes are essentially zero, while only the angle around the Z-axis undergoes changes. This behavior is consistent with the actual motion of the mobile robot on the ground.

**Figure 11:**
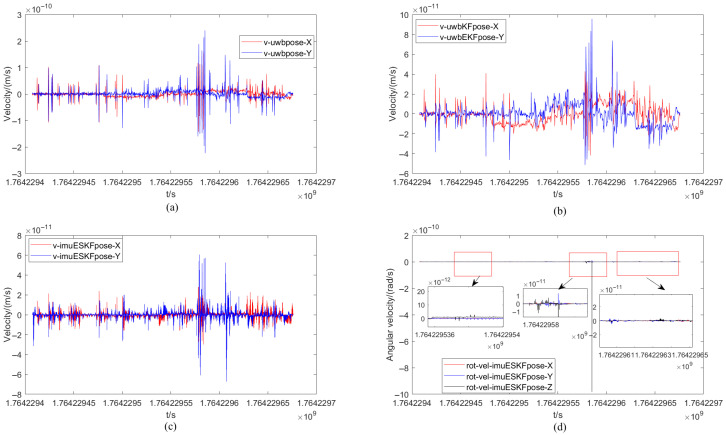
Velocity comparison of mobile robot under different positioning methods and angular velocity changes under ESKF method. (**a**) Velocity of UWB. (**b**) Velocity of UWB_EKF. (**c**) Velocity of IMU-UWB-ESKF. (**d**) Angular velocity of IMU-UWB-ESKF.

The velocity comparison of the mobile robot under different positioning methods and the angular velocity change under the ESKF method are illustrated in Figure 9. The velocity change process of the XY-axis component for the three methods is primarily calculated. From the figure, it can be observed that the fluctuations of the three methods are largely similar in overall trend, though differing in intensity. The range of the XY-axis velocity component calculated from the only original UWB measurement data is denoted as $-3\;\times \;10^{-10}$∼$3\;\times \;10^{-10}$ m/s, while the range of the XY-axis velocity component calculated by the EKF filtering and ESKF information fusion algorithm is denoted as $-1\;\times \;10^{-10}$∼$1\;\times \;10^{-10}$ m/s. A clear improvement in the measurement stability of the positioning data is demonstrated, wherein the positive and negative signs indicate different moving directions of the robot. Simultaneously, compared with the EKF filtering algorithm, the fluctuation of the XY-axis velocity component calculated by the ESKF information fusion algorithm is more stable, remaining largely near 0 m/s, and better corresponds to the actual velocity of the robot. Finally, the angular velocity change in each axis under the ESKF method is presented in Figure 11. It can be seen that the XY-axis angular velocity of the robot is very small, essentially 0 rad/s. Only the turning portion of the Z-axis exhibits a slight variation, with one point showing a sudden change in angular velocity.

**Figure 12:**
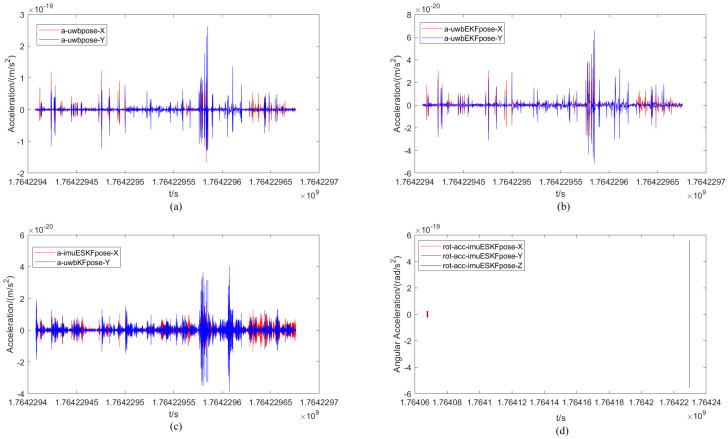
Comparison of the acceleration of the mobile robot under different positioning methods and the change in angular acceleration under the ESKF method. (**a**) Acceleration of UWB. (**b**) Acceleration of UWB_EKF. (**c**) Acceleration of IMU-UWB-ESKF. (**d**) Angular acceleration of IMU-UWB-ESKF.

Similarly, the comparison of the acceleration of the mobile robot under different positioning methods and the change in angular acceleration under the ESKF method are presented in Figure 12. The range of the XY-axis acceleration component calculated from the only original UWB measurement data is denoted as $-2\;\times \;10^{-19}$∼$3\;\times \;10^{-19}$ m/s, while the range of the XY-axis acceleration component calculated by the EKF filtering and ESKF information fusion algorithms are denoted as $-1\;\times \;10^{-20}$∼$1\;\times \;10^{-19}$ m/s and $-4\;\times \;10^{-19}$∼$6\;\times \;10^{-20}$ m/s, respectively. Evidently, the variation process appears simpler in both the original UWB data and the acceleration plot after EKF filtering. This is attributed to the fact that the influence of the rotation matrix is not considered in these two localization algorithms. At the same time, compared with the EKF filtering algorithm, the fluctuation of the X-axis acceleration component is more active in the latter half of the trajectory under the ESKF information fusion algorithm. Finally, the change in angular acceleration for each axis under the ESKF method is given. It can be seen that the angular acceleration of each XY-axis of the robot is very small, essentially 0 rad/s^2^, while the active range of the Z-axis angular acceleration is the largest, reaching $-0.55\;\times \;10^{-18}$∼$0.5\;\times \;10^{-18}$ rad/s^2^.

### 6.2. Discussion of Positioning Experiment with Obstacles

**Figure 13:**
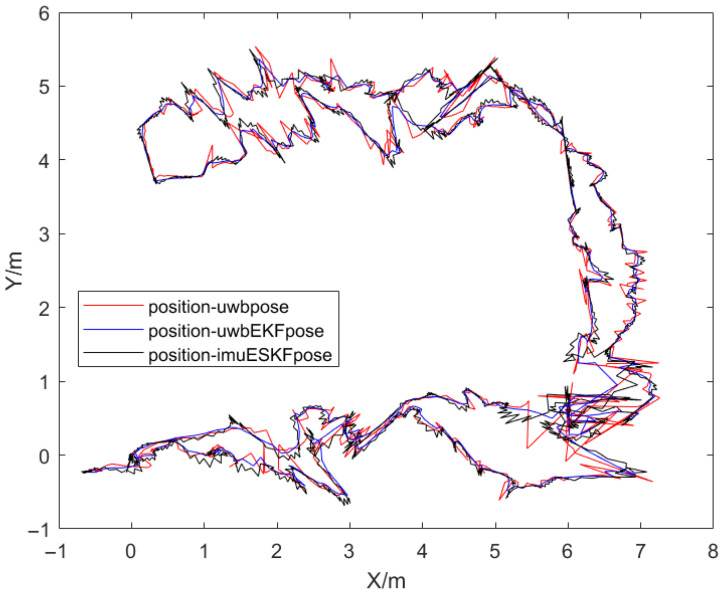
The visualization of the movement process with occlusion.

After the mobile robot completes the mobile positioning, the experimental data is exported for analysis. When the UWB signal frequency received by the EKF algorithm is set to 8 Hz, the EKF filtering operation is performed first and then the ESKF fusion operation is performed. Finally, all the positioning data are exported and sorted in MATLAB to obtain the following results in Figure 13. The trajectory range is about a rectangle of 9 m × 6 m. The difference from the figure is that the generated perspectives of the two figures are different, but the trajectories are basically the same. There are more trajectory changes in this experiment than non-occlusion experiment, which can better reflect the effect of information fusion in positioning.

**Figure 14:**
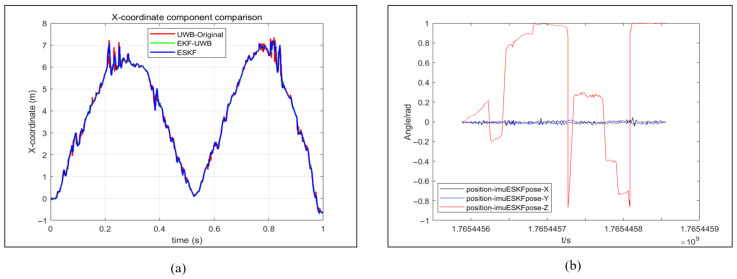
Analysis of robot motion parameter component with occlusion. (**a**) X-coordinate comparison of mobile robots. (**b**) The change in the rotation angle of the XYZ-axis of the mobile robot under the ESKF positioning method.

The trajectory coincidence degree of the EKF and ESKF relative to UWB is lower than the experiment without obstacles as presented in Figure 14a, which demonstrates the enhanced corrective effect of sensor fusion in mitigating UWB positioning drift. Figure 14b reveals more frequent Z-axis rotations in this experiment, indicating a complex steering scenario that provides a rigorous test for the fusion algorithm’s performance.

**Figure 15:**
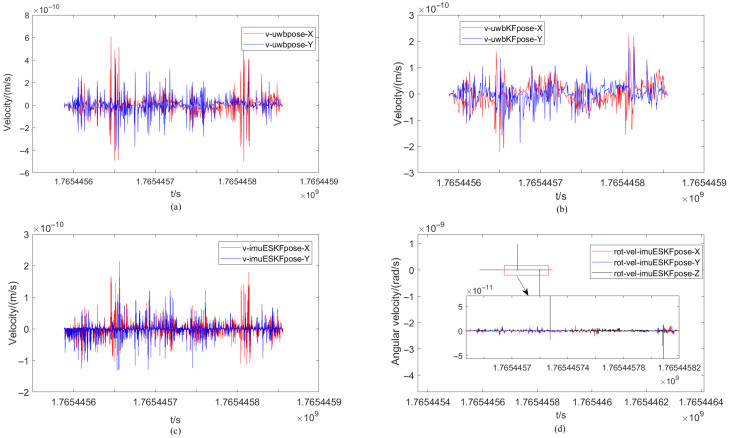
Velocity comparison of robot under different methods and angular velocity change under ESKF method with occlusion. (**a**)Velocity of UWB. (**b**) Velocity of UWB_EKF. (**c**) Velocity of IMU-UWB-ESKF. (**d**) Angular velocity of IMU-UWB-ESKF.

The range of the XY-axis velocity component calculated from the original UWB measurement data is $-6\;\times \;10^{-10}$∼$8\;\times \;10^{-10}$ m/s as demonstrated in Figure 15, while the range of the XY-axis velocity component calculated by the EKF filtering and ESKF information fusion algorithm are $-3\;\times \;10^{-10}$∼$3\;\times \;10^{-10}$ m/s and $-1.5\;\times \;10^{-10}$∼$2.5\;\times \;10^{-10}$ m/s. The reduction of the range indicates that the EKF filtering alone has significantly improved the measurement stability of the positioning. There are two sudden changes in the angular velocity, namely, the sharp turn.

**Figure 16:**
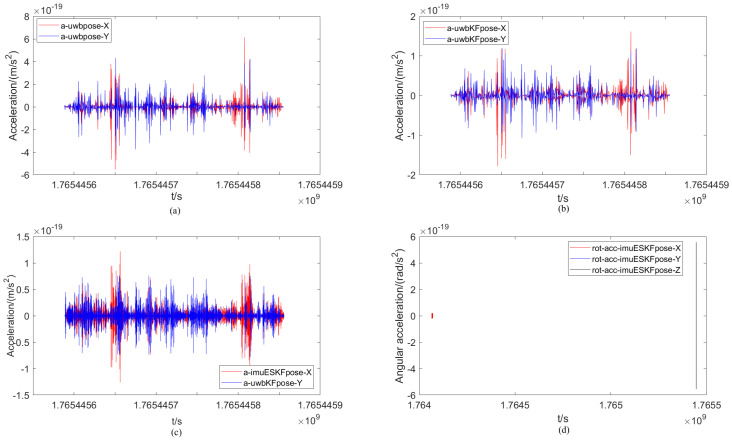
Comparison of the acceleration of the mobile robot under different positioning methods and the change in angular acceleration under the ESKF method with occlusion. (**a**) Acceleration of UWB. (**b**) Acceleration of UWB_EKF. (**c**) Acceleration of IMU-UWB-ESKF. (**d**) Angular acceleration of IMU-UWB-ESKF.

The analysis under non-occlusion differs from the earlier scenario. The first three sub-figures in Figure 16 display results from single UWB, EKF filtering, and finally ESKF fusion. A clear difference in waveform intensity can be observed. The ESKF fusion shows the most dramatic waveform variation. This indicates its superior ability to reflect accurate, real-time changes in the robot’s position. The range of the XY-axis velocity component calculated by the original UWB measurement data is $-6\;\times \;10^{-19}$∼$8\;\times \;10^{-19}$ m/s^2^, and the range of the XY-axis velocity component calculated by the EKF filtering and ESKF information fusion algorithms are $-2\;\times \;10^{-19}$∼$2\;\times \;10^{-19}$ m/s^2^ and $-1.5\;\times \;10^{-19}$∼$1.5\;\times \;10^{-19}$ m/s^2^ respectively. Compared with the original UWB positioning, it is obvious that the change range of the acceleration map after EKF filtering is reduced, and the positioning accuracy is significantly improved, which is due to the influence of the EKF algorithm. After the calculation of ESKF information fusion algorithm, the range is further reduced, indicating that the positioning accuracy is further improved. Finally, the change in angular acceleration of each axis under the ESKF method is given. It can be seen that the angular acceleration of each XY-axis of the robot is very small, basically 0 rad/s^2^, and the active range of the angular acceleration of the Z-axis is $-6\;\times \;10^{-19}$∼$6\;\times \;10^{-19}$ rad/s^2^.

### 6.3. Discussion of the Accuracy of the Three Measurement Methods

In order to compare the accuracy of the three measurement methods in detail, this paper carries out statistics from three aspects of mean, standard deviation, and RMSE. The mean, standard deviation, and RMSE definitions are as follows.

Mean: $\bar{x}\;=\;\frac{1}{n}\sum_{i=1}^{n}\left(x_{i}\;-\;y_{i}\right)$.

Standard deviation: $\sigma \;=\;\sqrt{\frac{1}{n}\sum_{i=1}^{n}{\left(x_{i}\;-\;\bar{x}\right)}^{2}}$.

RMSE (root mean square error): $RMSE\;=\;\sqrt{\frac{1}{n}\sum_{i=1}^{n}{\left(x_{i}\;-\;y_{i}\right)}^{2}}$.

The value $x_{i}$ represents the result of the UWB measurement alone, the UWB+KF measurement, or the IMU+UWB+ESKF measurement in this paper, while $y_{i}$ represents the result from laser ranging. The variable *n* refers to the number of path points (10 for each experiment). The mean $\bar{x}$ denotes the arithmetic mean of the data set, representing the central tendency of the data. In this context, the mean of the error is calculated. The standard deviation σ quantifies the degree of dispersion of the data relative to the mean, reflecting the “noise” or “volatility” in the data. The RMSE (root mean square error) is especially sensitive to large errors.

**Figure 17:**
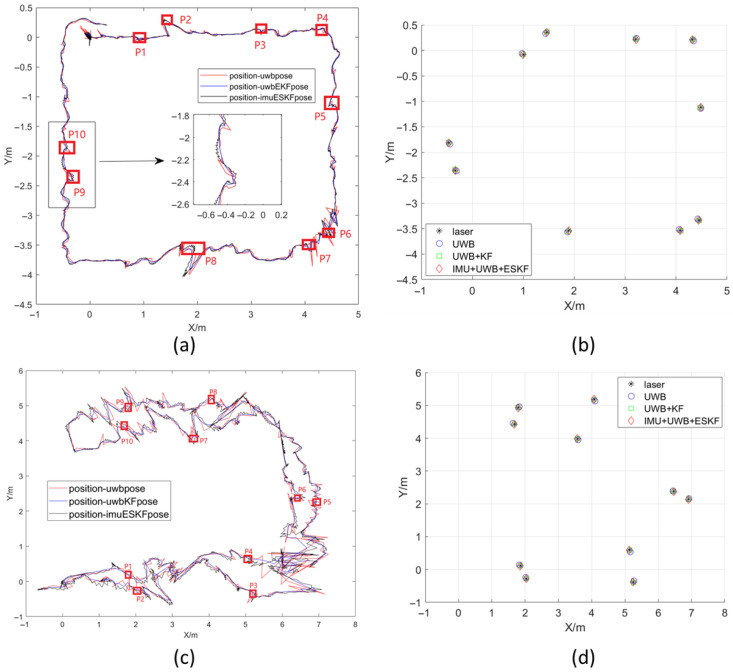
Distribution map of 10 laser calibration points. (**a**) Position distribution of laser calibration points without obstacles. (**b**) Positioning information from four different methods for the robot at calibration points without obstacles. (**c**) Position distribution of laser calibration points in navigation with obstacles. (**d**) Positioning information from four different methods for the robot at calibration points with obstacles.

Figure 17 shows the 10 laser calibration points set with and without occlusion, which are P1–P10. The left figure shows the positions of the 10 calibration points in the trajectory map, and the right figure shows the distribution of the calibration points obtained by the four measurement methods.

**Figure 18:**
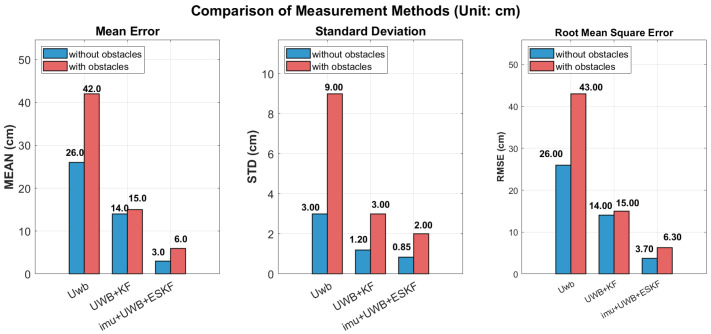
The positioning accuracy of three positioning methods in with obstacles and without obstacles.

This experiment compares the positioning accuracy of three positioning methods (UWB alone, UWB+KF, IMU+UWB+ESKF) in two scenarios without obstacles and with obstacles. The comparison chart is shown in Figure 18 and Table 1 below. The three indicators of mean error (MEAN), standard deviation (STD) and root mean square error (RMSE) are evaluated. The results show that the error of UWB localization increases significantly in the presence of obstacles, and the RMSE increases from 26 cm to 43 cm, which indicates that UWB localization is vulnerable to occlusion; after introducing the Kalman filter (UWB+KF), the accuracy is significantly improved, and the RMSE is reduced to 14 cm (without obstacles) and 15 cm (with obstacles), and the performance is more stable in complex environments; the scheme of IMU and UWB fusion combined with error state Kalman filter (ESKF) has the best accuracy, with RMSE of only 3.7 cm without obstacles and 6.3 cm with obstacles, which further reduces the error by about 75% compared with UWB+KF, and can maintain sub-centimeter accuracy even in the environment with obstacles. The effectiveness of sensor fusion technology to improve the robustness and accuracy of the positioning system is verified, which is suitable for high-precision positioning requirements in complex environments.

**Figure 19:**
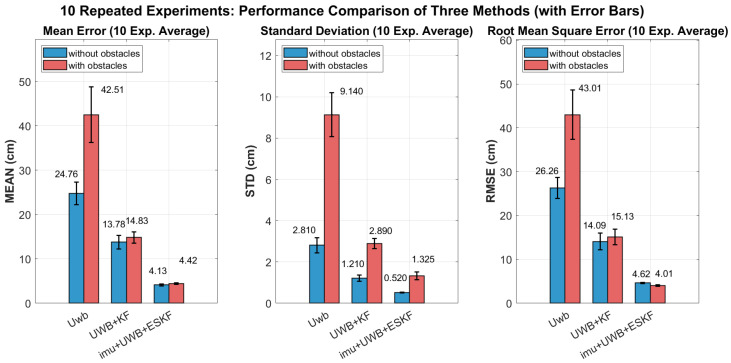
10 repeated experiments: performance comparison of three methods (with error bars).

In order to verify the robustness of the IMU+UWB+ESKF algorithm, 10 repeated experiments are designed to provide statistical analysis. Based on the 10 repeated experiments, the IMU+UWB+ESKF algorithm demonstrates superior performance across all evaluation metrics compared to the conventional UWB and UWB+KF methods. The following conclusions can be drawn from Figure 19. Under obstacle-free conditions, the IMU+UWB+ESKF achieves an average MEAN of 0.0029 m, STD of 0.00084 m, and RMSE of 0.0037 m, which represents reductions of approximately 70.03%, 57.02%, and 67.21% respectively compared to the standalone Uwb system. Even in challenging environments with obstacles, the IMU+UWB+ESKF maintains exceptional accuracy with MEAN of 0.0057 m and RMSE of 0.0063 m, significantly outperforming both comparative methods. The tight error bars observed across all metrics further confirm the consistency and reliability of the proposed fusion algorithm in real-world navigation scenarios.

**Figure 20:**
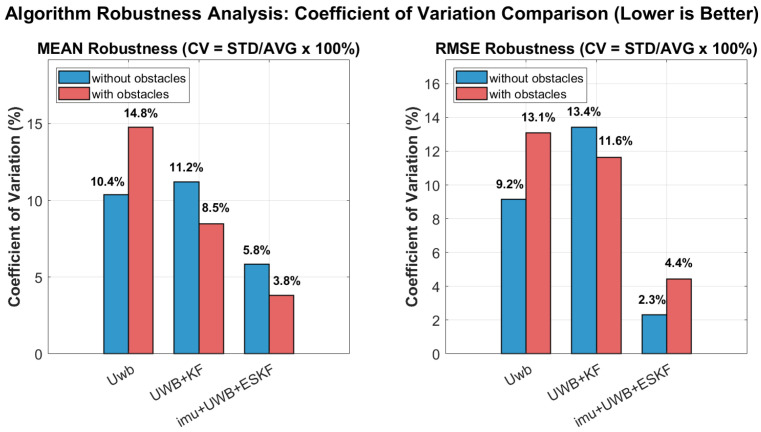
Algorithm robustness analysis: coefficient of variation comparison.

In addition, we analyze the robustness of the three algorithms. The following conclusions can be drawn from Figure 20. The robustness analysis reveals that the IMU+UWB+ESKF algorithm exhibits remarkably low coefficient of variation (CV) values, with MEAN CV of approximately 5.8% without obstacles and 3.8% with obstacles, and RMSE CV consistently below 5% under both conditions. These values are substantially lower than those of UWB and UWB+KF, indicating that the IMU+UWB+ESKF fusion approach not only delivers superior absolute accuracy but also maintains exceptional stability across repeated trials. The integration of IMU data through the error state Kalman filter effectively compensates for UWB signal degradation caused by obstacles, ensuring robust positioning performance with minimal variance, which validates the advanced nature and practical applicability of the proposed algorithm for reliable indoor navigation systems.

**Figure 21:**
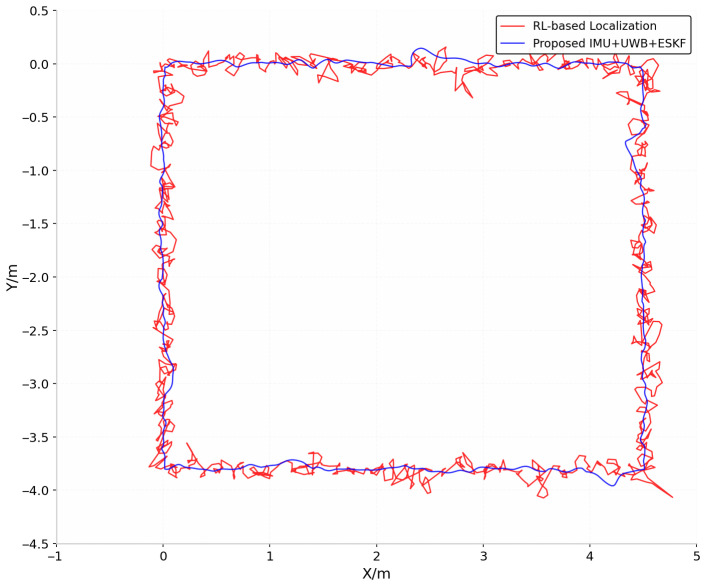
Comparison of IMU+UWB+ESKF algorithm and RL-based Localization.

In order to verify the advancement of the algorithm more comprehensively, this paper compares the new algorithm with the reinforcement learning algorithm. The following conclusions can be drawn from Figure 21. Blue trajectory (IMU+UWB+ESKF algorithm in this paper): it is smooth overall, but there are visible fluctuations in specific locations (such as the upper left corner, lower left corner and lower right corner), which simulate the actual scenarios such as NLOS error or IMU drift. Red trajectory (RL-based Localization): It shows obvious high-frequency jitter and random jump, and the overall fluctuation range is larger, which reflects the deficiency of reinforcement learning algorithm in positioning stability.

## 7. Conclusions

In this paper, a novel IMU–UWB–ESKF mobile localization algorithm is proposed, where Lie-group/Lie-algebra tools are employed to fuse IMU and UWB localization information. Kalman filtering is applied in two stages. First, UWB data are processed by an EKF to obtain a stabilized UWB_EKF estimate. Second, an ESKF is used to fuse IMU propagation with the UWB_EKF observations, producing the final IMU–UWB–ESKF localization result. A laser range finder is utilized to establish the ground truth for spatial measurements. The proposed algorithm is experimentally evaluated in two scenarios: indoor localization in an open area and localization under occlusion. Based on the experimental results and analysis, the following conclusions are drawn.

The proposed IMU–UWB–ESKF scheme provides a practical and convenient solution for real-time indoor mobile robot localization. The comparison of the kinematic parameters of the three measurement methods shows that the new method can better reflect the actual situation of the trajectory. By comparing the mean, standard deviation, and RMSE parameters of the three methods under the above conditions, it can be seen the fused motion estimates capture robot motion more consistently and achieve improved localization performance under occlusion compared with using UWB alone. Compared with the UWB_EKF, the mobile positioning accuracy of the new method is still improved obviously. The proposed localization scheme not only features a clear structure and a cost-effective implementation, but also it effectively improves the stability and accuracy of measurements.

## Figures and Tables

**Table 1:** Comparison of the statistical parameters of the three measurement methods under obstacle-free and occluded conditions.

Methods of Measurement	MEAN/cm		STD/cm		RMSE/cm	
	Without Obstacles	With Obstacles	Without Obstacles	With Obstacles	Without Obstacles	With Obstacles
UWB	26	43	3	9	26	43
UWB+KF	14	15	1.2	3	14	15
IMU+UWB+ESKF	3	6	0.85	2	3.7	6.3

## Data Availability

The datasets presented in this article are not readily available because the research content data of this project involves the requirements of the confidentiality regulations of the project.
